# Word encoding during sleep is suggested by correlations between word-evoked up-states and post-sleep semantic priming

**DOI:** 10.3389/fpsyg.2014.01319

**Published:** 2014-11-14

**Authors:** Simon Ruch, Thomas Koenig, Johannes Mathis, Corinne Roth, Katharina Henke

**Affiliations:** ^1^Department of Psychology, University of BernBern, Switzerland; ^2^Center for Cognition, Learning and Memory, University of BernBern, Switzerland; ^3^Department of Psychiatric Neurophysiology, University Hospital of Psychiatry, University of BernBern, Switzerland; ^4^Department of Neurology, Inselspital, Bern University Hospital, University of BernBern, Switzerland

**Keywords:** unconscious, implicit, memory, semantic priming, NREM sleep, slow-oscillations

## Abstract

To test whether humans can encode words during sleep we played everyday words to men while they were napping and assessed priming from sleep-played words following waking. Words were presented during non-rapid eye movement (NREM) sleep. Priming was assessed using a semantic and a perceptual priming test. These tests measured differences in the processing of words that had been or had not been played during sleep. Synonyms to sleep-played words were the targets in the semantic priming test that tapped the meaning of sleep-played words. All men responded to sleep-played words by producing up-states in their electroencephalogram. Up-states are NREM sleep-specific phases of briefly increased neuronal excitability. The word-evoked up-states might have promoted word processing during sleep. Yet, the mean performance in the priming tests administered following sleep was at chance level, which suggests that participants as a group failed to show priming following sleep. However, performance in the two priming tests was positively correlated to each other and to the magnitude of the word-evoked up-states. Hence, the larger a participant's word-evoked up-states, the larger his perceptual and semantic priming. Those participants who scored high on all variables must have encoded words during sleep. We conclude that some humans are able to encode words during sleep, but more research is needed to pin down the factors that modulate this ability.

## Introduction

Although sleep is a state of reduced consciousness, we still process sensory information while asleep. The sleeping brain responds differently to semantically congruent vs. incongruent sentences (Daltrozzo et al., 2012), to semantically related vs. unrelated words (Brualla et al., 1998), and to one's own name vs. names of others (Perrin et al., 1999) reflecting word understanding. If words are understood during sleep (Brualla et al., 1998; Perrin et al., 1999; Daltrozzo et al., 2012), we speculate that they might be stored as well which might alter subsequent processing of these words due to priming. But sound evidence for this claim is missing (see Aarons, 1976; Eich, 1990; Simon and Emmons, 1955; Hoskovec, 1966 for reviews on sleep-learning studies). So far, there are only reports of non-verbal learning during sleep (Ikeda and Morotomi, 1996; Arzi et al., 2012; Hauner et al., 2013). For example, Arzi et al. (2012) showed that humans take deeper breaths if they hear a tone that was repeatedly associated with a pleasant odor during sleep. Hauner et al. (2013) further demonstrated extinction learning during sleep if slumbering participants are re-exposed to an odor that was used as context odor during a contextual fear conditioning task. Tone-odor conditioning and extinction learning do not involve processing of conceptual information and might mainly rely on subcortical and hippocampal networks. Evidence of verbal learning during sleep would demonstrate that the sleeping brain is also able to acquire abstract conceptual knowledge whose processing depends on the neocortex.

The success of word encoding during sleep might depend on the sleep stage, during which words are presented. Although rapid eye-movement (REM) sleep is characterized by dreaming (Siclari et al., 2013) and wake-like cerebral activity, non-rapid eye-movement (NREM) sleep might actually provide better conditions for word encoding due to its role in memory consolidation (Diekelmann and Born, 2010; Rasch and Born, 2013). NREM sleep consists of stage 1 sleep (S1) that builds the transition between wakefulness and sleep, stage 2 (S2) or light sleep, and deep sleep stages 3 and 4 (S3/S4) which are often referred to as slow-wave sleep (SWS). S2 sleep is characterized by the presence of spindles and k-complexes in the electroencephalogram (EEG). Sleep spindles are brief oscillatory events at frequencies between 9 and 16 Hz. They are most frequent during S2 but are also present in SWS. K-complexes are sharp negative signal deflections followed by a slower positive component. They bear much similarity with the high-amplitude, slow-frequency (<4 Hz) activity that characterizes the deep NREM sleep SWS. In SWS, EEG activity is dominated by slow-oscillations with peak frequencies at about 0.8 Hz (Mölle et al., 2002). These oscillations are generated in neocortical neurons which slowly and synchronously alternate between phases of membrane depolarization accompanied by increased firing (up-states), and phases of membrane hyperpolarization with reduced firing (down-states) (Steriade et al., 1993; Mölle et al., 2002). Slow-oscillations and especially up-states are thought to play a vital role in memory consolidation. Much evidence suggests that memories formed during the day are reactivated and thereby strengthened during NREM sleep (Diekelmann and Born, 2010; Rasch and Born, 2013). Reactivation benefits from the increased neuronal activity and excitability that is provided by up-states. Up-states are thought to boost neuronal plasticity, which helps to strengthen reactivated memories. We hypothesize that the activity and plasticity provided by neocortical up-states might also assist word encoding.

We played single words in a rhythmic manner while participants were in deep NREM sleep during an afternoon nap. Several studies suggested that the rhythmic presentation of sounds during NREM sleep entrains slow-oscillations such that up-states start to occur regularly upon sound presentation (Ngo et al., 2013a,b). These investigators observed up to two entrained slow-oscillations within 3 s upon sound presentation. To investigate whether slow-oscillations are similarly entrained by rhythmically played words, we analyzed event-related EEG activity within the 3 s following word onset. We correlated the size of the word-evoked up-states with performance in the two priming tests that were administered following the nap to find out whether entrained up-states contribute to word priming. Because up-states provide windows of increased neocortical plasticity, we hypothesized that large word-evoked up-states would benefit word encoding. Following the nap, we administered two behavioral tests that assessed priming from sleep-played words. We hypothesized that successful encoding of words during sleep would improve the subsequent processing of these and semantically related words due to priming. Priming is thought to be a by-product of the modification of existing knowledge (Bowers, 2003; Marsolek, 2003) and thus reflects a simple form of learning. Because priming effects are long-lived (Tulving et al., 1982; Woltz and Shute, 1995) and even observed in amnestic patients with impaired explicit memory (Carlesimo, 1994), tests of priming are suitable to look for an imprint that sleep-played words leave on memory.

## Materials and methods

### Participants

We tested 26 healthy men who reported normal sleep routines. Participation was compensated with 90 Swiss Francs (~90$). Ten men were excluded after data collection because of poor polysomnography (*n* = 2) or because less than 2/3 of the planned stimuli could be presented during NREM sleep due to insufficient sleeping time (*n* = 8). We report the data of the remaining 16 participants (age: mean ± *SD* = 23.75 ± 2.44 years). The study was introduced as an investigation of the effect of noise on sleep quality to keep participants naïve to word presentations during sleep. This was necessary to promote unconsciousness of word presentation. Written semi-informed consent was obtained from all participants before experimentation. The fact that words were played during sleep was revealed following sleep. The study was approved by the local ethics committee “Kantonale Ethikkommission Bern (KEK),” in Bern, Switzerland and was conducted in accordance with the guidelines of the Declaration of Helsinki.

### Procedure

Participants were instructed to sleep from 2 to 7 a.m. and to abstain from caffeine on the day of testing to promote sleep during the afternoon nap. Actigraphy and semi-structured interviews indicated good compliance with these instructions. Upon arrival at the sleep laboratory, participants were prepared for polysomnography. Next, we assessed whether the minimal volume defined for stimulation during sleep was within participants' hearing range. Based on a pilot study, we used a preset volume of 53.2 dB(A) for presenting words during sleep. If this preset volume disturbed an individual's sleep, we reduced the volume to levels above the lower bound of 47.2 db(A). To confirm that this lower bound was within hearing range, we measured the minimal volume at which each participant could detect pseudowords embedded in background noise (noise that was later played during sleep). This assessment was introduced as a hearing test. The minimal volume was the point where the presence of noise-embedded pseudowords could just be detected in 98% of presentations. This volume ranged between 36.2 and 42.6 dB(A) and was below the lower bound of 47.2 db(A).

At 1.30 p.m., participants took their nap. They were not informed of sleep-played words. Throughout the entire nap a pleasant mixture of brown and pink noise was playing at an unobtrusive volume of 56.6 dB(A). This background noise should reduce the salience of words and should thereby promote unconsciousness of word encoding. When participants entered stable NREM sleep, as indicated by visible slow-wave (1–4 Hz) activity at frontal electrodes during at least 30 s, word presentation was initiated. We started by presenting pseudowords at a very low volume level to habituate participants to noise-embedded words. Then, the presentation volume was increased to 53.2 dB(A) and the words were presented. Word presentation was stopped if an arousal (evoked by various reasons) appeared in the sleep-EEG. The average number of stops per participant was 4.75 ± 5.79 (*SD*). In case of repeated arousals upon word presentation, we lowered the volume from 53.2 dB(A) but kept it above the lower bound of 47.2 dB(A). Consequently, volumes varied both within participants [range: 47.2 dB(A)–53.2 dB(A)] and between participants [range of average volume: 50.2 dB(A)–53.2 dB(A)].

A total of 56 nouns were presented during NREM sleep. Half were played once and half six times to boost sleep-learning through repetition. Yet, some participants' sleep was too short to present the complete set of words. In several of these participants the number of available trials was too low to reliably assess differences between once and repeatedly played words. Therefore, we pooled data over conditions (see Statistical Analyses). We presented words intermixed between conditions avoiding the immediate repetition of a word. Participants were wakened, when either all words were presented or when they failed to fall back into stable NREM sleep after an arousal. The 16 analyzed participants spent on average 14.87 min in slow-wave sleep (SWS) and 23.31 min in sleep stage 2 (S2). We aimed at word learning mainly during SWS: Of 178.63 presentations during NREM sleep (*SD* = 23.27, range: 124–196) as much as 154.44 (*SD* = 41.88) presentations were conducted during SWS and only 24.19 (*SD* = 34.4) during S2.

#### Semantic priming test

**Figure 1:**
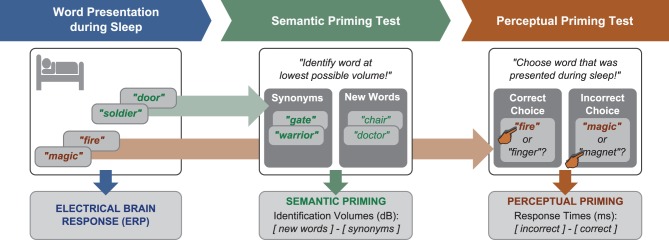
**Design**. Word presentation during sleep was followed by two priming tests. In the semantic test, identification volumes for synonyms to sleep-played words and new words were contrasted. In the perceptual test, response latencies for correct and incorrect responses in a two-alternative forced-choice recognition test for sleep-played words were compared.

Following waking, participants were given a break of 15 min to recover from sleep inertia. Then, we administered a semantic priming test to find out whether participants would show priming of the meaning of sleep-played words (Figure 1). This test recorded the minimal volume at which participants identified synonyms to sleep-played words vs. new words (control condition). If the sleeping brain had encoded and stored the meaning of a word like “soldier,” it should be prepared to identify a semantically related word like “warrior” more easily—i.e., at lower volumes—than a new word. This test was introduced as a hearing test. The background noise that played during sleep kept playing at 56.6 dB(A) during this test. On each trial, a word was repeatedly presented with increasing volume until the participant could identify it. Identification was signaled by button press and the pronunciation of the heard word. Only correctly identified words went into analysis. Each word was initially presented with a volume of 38.7 dB(A). This volume was increased with each word repetition by 2.5 dB(A). The average identification volume was 53.7 dB(A). Of the 56 words played for identification, 14 were synonyms to one-fold and 14 to six-fold sleep-played words. Another 28 words were new and belonged to the control condition. Words were presented intermixed between conditions. The difference between the mean identification volume for new words (higher) and synonyms (lower due to priming) was the score of semantic priming. We chose this priming test because Stuart and Jones (1995, 1996) had found that this test is sensitive to perceptual priming. Before the main experiment, we had carried out a pilot study to make sure that the task is also susceptible to semantic priming. In the encoding part of this pilot study, 19 participants performed an auditory attention task during which prime words were played in the background for incidental encoding. Incidental encoding was used to mimic word-encoding during sleep. The attention task required participants to indicate on which ear a repeatedly playing beep tone was presented. This task ensured that participants focused on the auditory modality. Each prime word was played ten times in sequence during a 6 s encoding episode. Following each encoding episode, either a synonym of the prime word or a semantically unrelated new word was presented as target in the word identification task. Participants identified synonyms to played prime words at lower volumes than unrelated words [mean and s.e.m. of volume difference: 0.87 ± 0.31 dB(A), *t*_(18)_ = 2.77, *p* = 0.01, *r* = 0.55]. Hence, this test was considered adequate for our sleep study.

#### Perceptual priming test

Next, we administered a test of perceptual priming to find out whether participants remembered sleep-played words explicitly (consciously) or implicitly (unconsciously). Before we applied this test, we informed participants that words were embedded in the noise that played during sleep. All participants were surprised to hear this and assured that they were not aware of any words. The perceptual retrieval test should provide a more objective measure of word awareness during sleep, namely forced-choice accuracy. On each of 28 trials, participants were played with normal volume either a one-fold or a six-fold sleep-played word plus a new word to decide which word had been presented during sleep (answer by button press). The order of the two words in a pair was randomized. Participants were forced to guess because they were not aware of sleep-played words. The percentage of correct choices served as an objective measure of word awareness during the nap. The reaction times for correct and false responses served as an implicit measure that reflects perceptual word priming. Perceptual priming was hypothesized to reflect in the difference between the mean reaction latency for incorrect minus correct choices. Previous studies had suggested that participants respond faster when correctly retrieving unconsciously learned information (e.g., Henke et al., 2003; Duss et al., 2011). A positive difference score between incorrect minus correct responses was thus assumed to reflect perceptual priming of sleep-played words. The percept may be the sound or rhythm of the spoken word.

### Hardware and software

We used the software Presentation® (14.5, Neurobehavioral Systems, http://www.neurobs.com) for stimulus delivery. Sounds were produced using the audio interface Audio 2 DJ by Native Instruments (http://www.native-instruments.com) and in-ear headphones CX300II by Sennheiser. The EEG was recorded with the amplifier N7000™ and the software Somnologica Studio Version 5.1.1 by Embla (http://www.embla.com/).

### Stimuli

We presented two-syllabic German nouns during sleep. Playing only two-syllabic words was assumed to help create a rhythmic auditory stimulation pattern that entrains slow-oscillations. The retention of half of sleep-played nouns was assessed in the semantic priming test and the retention of the other half in the perceptual priming test. In the semantic priming test, targets were synonyms to sleep-played nouns and distracters were semantically unrelated nouns. In the perceptual priming test, targets were the sleep-played nouns and distracters were also semantically unrelated nouns. The procedure for creating the stimuli for the two priming tests was the following: we formed word triplets that consisted of a two-syllabic sleep-played prime word, a synonym of varying word length and syllable count for the semantic test, and a semantically unrelated, frequency-matched two-syllabic distracter noun for the perceptual test. These word triplets were assigned to six lists (A–F) of 14 triplets each (all words and data on word-frequency, duration of spoken words, syllable counts of synonyms, and word concreteness are presented in the Supplementary Table 1). Four lists (e.g., A-D) were used to play prime words during sleep. The primes and distracter nouns of two of these lists (e.g., A, B) were later used for the perceptual priming test. The synonyms of the other two lists (e.g., C, D) were used for the semantic priming test. Because synonyms varied regarding word length across word triplets, whereas the primes and distracters were always two-syllabic, we used the synonyms of prime words from the two remaining lists (e.g., E, F) that were not presented during sleep as new words (distracters) in the control condition of the semantic priming test. This guaranteed that word length was similarly distributed in targets (synonyms two sleep-played nouns) and distracters. Lists were counterbalanced over tasks between participants, such that each list was equally often used for the perceptual and the semantic test. Furthermore, primes and distracters were interchanged in half of the instances where a specific word list was used for the perceptual priming task. For the semantic priming task, primes and synonyms were never interchanged because synonyms varied regarding syllable count, whereas primes were always two-syllabic.

The six stimulus lists were matched for word-frequency, duration of spoken words, syllable count, and concreteness. Supplementary Tables 2–5 provide descriptive statistics for these variables per word list and word type (prime, synonym, distracter). We used logarithmized frequency data provided by the Leipzig Wortschatz Lexicon (http://corpora.informatik.uni-leipzig.de/) to match lists regarding word frequency. Syllable counts were also drawn from the Leipzig Wortschatz Lexicon. Two raters classified concreteness of words on a binary scale (100% rater agreement). The time from word onset to offset in the recorded sound files was the duration of spoken words.

Word lists differed neither regarding word frequency nor word duration nor syllable count nor concreteness [all *F*_(5, 78)_ < 1.12, all *p* > 0.36, all η^2^_*p*_ < 0.07]. This was suggested by separate ANOVAs run for each matching criterion (frequency, duration, concreteness) and each word type (primes, synonyms, distracters) where word list was entered as sole factor. Furthermore, prime and distracter words were statistically equal regarding word frequency, word duration, and concreteness. This was tested with separate repeated-measures ANOVAs for each matching criterion where word-type (prime vs. distracter) was entered as within- and word-list as between-subjects factor. Measures of frequency, duration, and concreteness were equal between word-lists [all *F*_(5, 78)_ < 0.15, *p* > 0.98, η^2^_*p*_ > 0.01], word-types [all *F*_(1, 78)_ < 1.92, *p* > 0.17, η^2^_*p*_ < 0.02], and combinations of word-lists and word-types [all *F*_(5, 78)_ < 0.88, *p* > 0.50, η^2^_*p*_ < 0.05 for the word-list X word-type interaction].

The semantic relatedness between primes and their synonyms was assessed in two pilot studies using priming tests that differed from the test used in the main experiment. In both studies, 19 participants listened to prime words while performing an auditory attention task. Each prime word was played ten times in sequence during a 6 s encoding episode. Each encoding episode was followed by a word identification task (see semantic priming test) in pilot study 1, and an auditory lexical decision task in pilot study 2. The critical variable in the word identification task was the stimulus volume at which participants could identify synonyms to prime words vs. distracters. Primed synonyms were identified at lower volumes than unrelated words [mean and s.e.m. of volume difference: 0.87 ± 0.31 dB(A), *t*_(18)_ = 2.77, *p* = 0.01, *r* = 0.55] reflecting semantic priming. In the lexical decision task, encoding of a prime word was followed by the presentation of either a synonym to the prime word or a distracter. Correct classification was faster for synonyms vs. distracters [mean and s.e.m. of difference in response latencies: 34.70 ± 11.22 ms, *t*_(18)_ = 3.09, *p* < 0.01, *r* = 0.59]. Both pilot studies confirm that the semantic relatedness between primes and their synonyms is behaviorally relevant.

All words were spoken by a female voice and were recorded using professional audio equipment. Sounds were edited and volume-level normalized using the software Audacity® (http://audacity.sourceforge.net/). The duration of sleep-played words ranged between 0.45 and 0.95 s (mean = 0.65 s, *SD* = 0.1 s).

### Polysomnography and sleep scoring

Sleep was monitored by standard polysomnography (Iber et al., 2007) which included electroencephalography (EEG), electrooculography, electromyography, and electrocardiography. The EEG was recorded at 500 Hz and was obtained from F3, Fz, F4, C3, Cz, C4, P3, Pz, P4, O1, Oz, O2, with Fpz as ground and contralateral mastoids as reference electrodes. Sleep scorings were carried out according to Rechtschaffen and Kales (1968) by three scorers, who were blinded to periods where words were presented.

### EEG analysis

Only EEG data that were scored as NREM sleep were analyzed. Data segments that contained arousal, which consists of an abrupt shift to high frequency activity (Iber et al., 2007), or motor and other artifacts were visually identified and excluded from data analysis. If a word was played in the 3s-period before a rejected EEG segment, it did not enter EEG and behavioral data analysis. If a word belonging to the “word repetition” condition was excluded, we also excluded the EEG response to this word's repetition at other time points during sleep. The selected unfiltered EEG data were re-referenced to pooled mastoids and were segmented into epochs ranging from 1 s before to 3 s following word onset.

To analyze event-related potentials (ERPs), each epoch was baseline-corrected by subtracting the mean voltage of the 1 s pre-stimulus time period. We used 1 s pre-stimulus period—rather than a shorter period—for baseline correction in order to obtain signals that are unaffected by the current phase of the large-amplitude 1 Hz frequency, which dominates deep NREM sleep. The baseline-corrected epochs were averaged per participant.

To analyze event-related changes in spectral power (ERSPs), the data of each epoch were decomposed into the time-frequency domain using the function *newtimef()* by EEGLAB. We used sinusoidal wavelet transforms with 3 cycles in length at the lowest frequency of 4 Hz, increasing linearly with frequency to 20.0625 cycles at the highest frequency of 53.5 Hz. The time window was 834 ms. The decomposition produced a linear frequency space with 100 frequencies ranging from 4 to 53.5 Hz, and a time space with 200 time points ranging from -582 to 2582 ms. The raw power values at each time-frequency point for each epoch were divided by the mean raw power of the same frequency averaged across the entire epoch. The resulting relative power values were baseline corrected by subtracting the mean relative power of the prestimulus time window (−582 to 0 ms) of the corresponding frequency. These baseline-corrected epochs were then averaged per participant. Preprocessing was performed with the Matlab® toolbox EEGLAB (http://sccn.ucsd.edu/eeglab/) by Delorme and Makeig (2004).

We used a cluster-based non-parametric approach as proposed by Maris and Oostenveld (2007) to find peaks where the event-related signal (ERP) and spectral power (ERSP) deviated significantly from the pre-stimulus baseline. In brief, we (1) computed *t*-tests against the baseline (i.e., zero) for each time point or time-frequency point and each electrode. Next, we (2) identified coherent points in time-electrode space or time-frequency-electrode space, where all *t*-values were significant at the 5% level. For each cluster of connected points, the cluster mass (3) was then computed as the sum of the *t*-values of all included points. To test whether a specific cluster reflected a significant deviation from baseline, we compared its cluster mass with the permutation distribution, i.e., the distribution of the mass of clusters obtained from randomly permuted data sets. We obtained the permutation distribution by repeatedly permuting the data. To this aim, we randomly interchanged the evoked signal and the baseline in arbitrarily selected participants. We finally extracted the cluster mass of the largest cluster by performing steps (1) through (3) with each permuted data set. The 5000-fold repetition of this procedure yielded a valid estimation of the permutation distribution of the maximal mass of clusters in random data sets. The *p*-value of a specific cluster in the original data set was computed as the percentage of clusters in the permutation distribution that had the same or a larger mass. A cluster was accepted as a significant peak, if less than 5% of the clusters in the permutation distribution exhibited an equal or larger mass. The resulting peaks reflect deviations in the EEG that must have been evoked by played words.

We validated the results of this analysis for the ERP data by running a topographic consistency test (Koenig and Melie-García, 2010; Koenig et al., 2011). This test produced qualitatively similar results.

To assess whether word evoked brain responses during sleep predicted subsequent word priming, we correlated the magnitude of evoked ERPs with participants' performance scores attained in each priming test. ERP magnitude was computed separately for each participant and each cluster by averaging the amplitude (μV) across all points in time-electrode space for the respective significant cluster.

**Figure 2:**
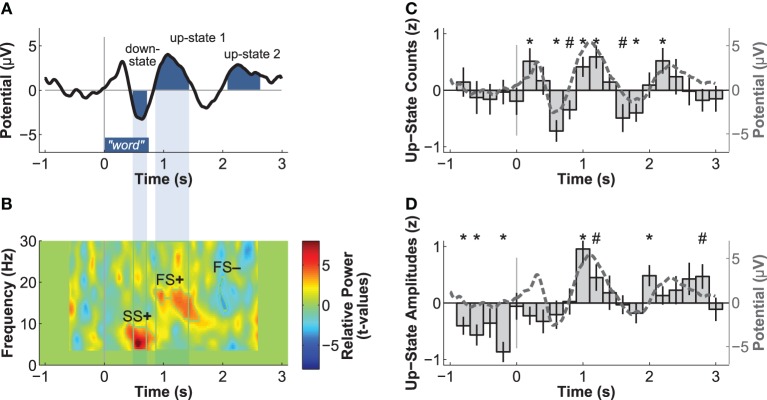
**Electric brain responses at electrode Fz to word presentation during sleep**. Event-related potentials **(A)** and event-related spectral power changes **(B)** to word-presentation during sleep. Temporal overlaps of the down-state with the increase in slow-spindle power (SS+) and of the up-state 1 with the increase in fast-spindle power (FS+) are highlighted. Normalized count **(C)** and peak-amplitudes **(D)** of discrete slow-oscillation up-states identified before and after word presentation. Both the number and the amplitudes of up-states are increased during the time windows of the ERPs identified as up-states 1 and 2, which indicates that these ERPs reflect up-states. ^*^p < 0.05, ^#^*p* < 0.10 (uncorrected *p*-values for two-tailed *t*-tests).

The next aim was to determine whether the occurrence of slow-oscillations was influenced by word presentation. We identified discrete slow-oscillations in the frontal electrode Fz using an algorithm of Mölle et al. (2009). Slow-oscillations detected with this algorithm had been found to be relevant to memory consolidation (Ruch et al., 2012). We applied a low-pass filter at 30 Hz, down-sampled the data to 250 Hz, and then applied a band-pass filter of 0.15–2 Hz. All epochs of the residual data that contained two consecutive positive-to-negative zero-crossings were selected as potential slow-oscillations. We computed the local minimum (negative peak) and maximum (positive peak) per epoch. An epoch was accepted as a discrete slow-oscillation, if (1) it had a duration between 0.9 and 2 s, (2) the amplitude of the negative peak was more negative than 2/3 of the average amplitude of all negative peaks, and (3) the difference between the amplitudes of the positive and negative peak was larger than 2/3 of all amplitude differences. To analyze the temporal association between word presentation and slow-oscillations, we counted the number of up-states (positive peaks) of discrete slow-oscillations occurring within the 0.2 s time bins from 1 s before to 3 s after stimulus onset. These counts were first z-transformed per participant and then averaged over participants (Figure 2C). We further averaged the z-transformed peak amplitudes of these up-states per time-bin to analyze the modulation of up-state amplitudes by word presentation (Figure 2D).

Assuming that word-evoked brain responses reflect entrained slow-oscillations, we compared the topographies of evoked responses with the topographies of spontaneously occurring slow-oscillations. For the evoked brain responses, individual amplitudes at the peak time of the averaged ERPs (see Table 1) were normalized by the subject's mean and standard deviation. For spontaneous slow-oscillations, we averaged the EEG data of all discrete slow-oscillations centered around the transition from down- to up-states for each participant. Amplitudes at the down-state peak (196 ms before the transition to the up-state) and up-state peak (176 ms after the transition to the up-state) were normalized by the subject's mean and standard deviation of the respective topography. The resulting normalized topographies were contrasted using repeated-measures ANOVAs with the two within-subject factors Topography (reflecting the 12 electrodes) and Event (evoked ERPs vs. spontaneous up-state or down-states).

**Table 1:** **Descriptive statistics for the event-related potentials that were evoked by words presented during sleep**.

**Response**	**Duration (ms)**			^*^p-Value	**Locus of maximal deviation from baseline**		
	**Start**		**End**		**Electrode**	**Peak *t*-value**	**Peak time**
Down-state	452	–	806	0.032	C4	−9.974	670
Up-state 1	868	–	1668	<0.001	P4	6.858	1168
Up-state 2	2078	–	2672	<0.001	P4	6.958	2400

* *p-values are based on non-parametric statistics as suggested by Maris and Oostenveld (2007)*.

### Statistical analysis

Data of the two conditions (one-fold vs. six-fold word presentation) were pooled for all statistical analyses to gain more statistical power. Data pooling was necessary because some participants slept too short to present them with the complete set of words (i.e., loss of trials). Data pooling was acceptable because word repetitions did not seem to modulate the word-evoked EEG responses nor priming performance. Due to the small sample size, non-parametric Spearman correlation coefficients *r_s_* are reported. Note that Pearson coefficients and the corresponding significance levels were very similar and are therefore not reported. We computed two-tailed *p*-values for all reported correlations and *t*-tests. For *t*-tests, r is reported as measure of effect size.

## Results

### Word processing during sleep

**Figure 3:**
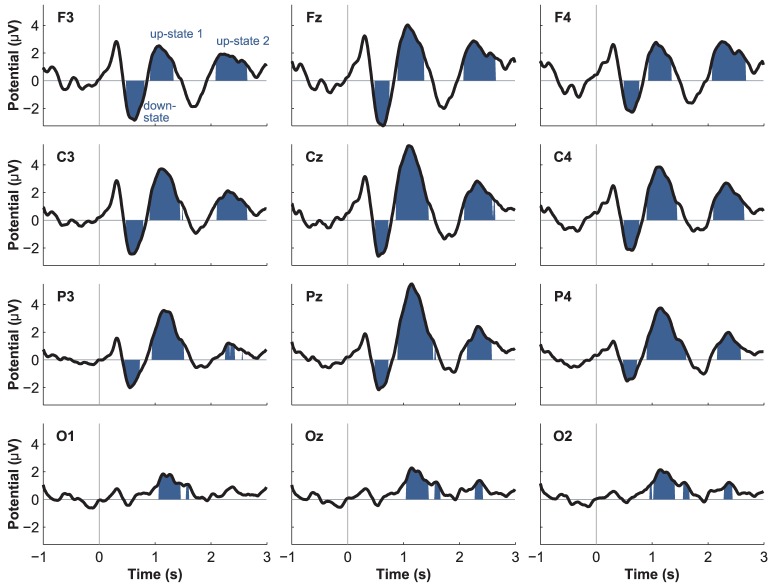
**Event-related brain potentials (ERPs) to word presentation during sleep**.

Event-related potentials (ERPs) to word presentation during NREM sleep indicated that participants noted words while asleep (Figure 2A). Words elicited a negative ERP around 500 ms and two positive ERPs at 1000 and 2400 ms following word-onset (all *p* < 0.05; see Table 1 for descriptive statistics and Figure 3 for plots of the ERPs at all recorded electrodes). We interpret these EEG responses as down-states (at 500 ms) and up-states (up-state 1 and 2 at 1000 and 2400 ms) of entrained slow-oscillations. Although slow-oscillations occur spontaneously during NREM sleep, they can be entrained by rhythmic presentations of sounds (Ngo et al., 2013a) such that they start to consistently appear following sounds. We suggest that the rhythmic presentation of words entrained a sequence of two slow-oscillations with down-states at 500 ms and up-states at 1000 and 2400 ms following word onset. Up-states were of special interest to us because they provide brief windows of increased neocortical excitability (Bergmann et al., 2012; Schabus et al., 2012) that promote neocortical processing such as the consolidation of previously formed memories (Ngo et al., 2013b). The word-entrained up-states in our study reflect sleep-specific brain states that might assist the initial word processing or the ensuing consolidation of the sound and meaning of the encoded words.

The analysis of automatically identified discrete slow-oscillations (Mölle et al., 2009) confirmed that the ERPs at 1000 and 2400 ms reflect entrained up-states. Discrete up-states occurred more frequently and with higher peak-amplitudes at around 1000 and 2400 ms following word-onset (Figures 2C,D). The number of up-states occurring per second was significantly elevated during the time windows of the ERPs at 1000 ms [mean ± s.e.m.: 0.450 ± 0.011 up-states/s; *t*_(15)_ = 4.34, *p* < 0.001, *r* = 0.75] and 2400 ms [0.464 ± 0.019 up-states/s; *t*_(15)_ = 4.17, *p* < 0.001, *r* = 0.73] when contrasted with the ERP at 500 ms (0.343 ± 0.020 up-states/s).

**Figure 4:**
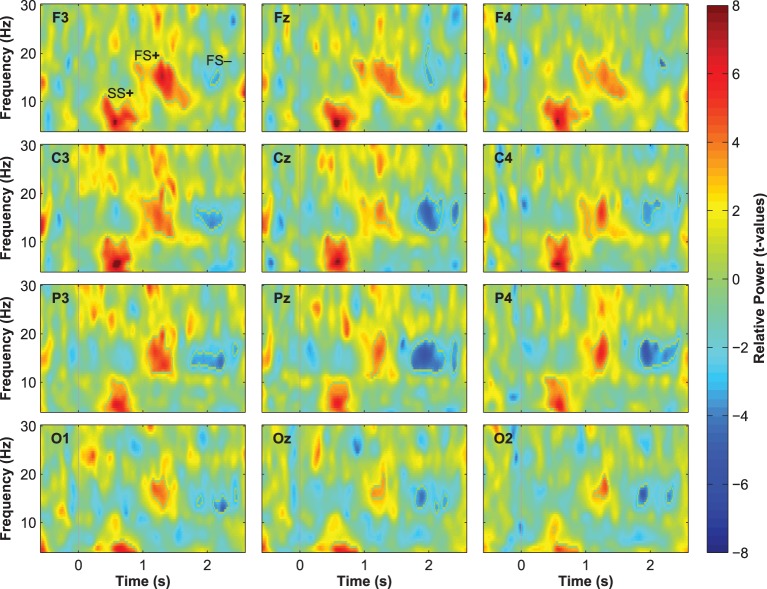
**Event-related changes in spectral power (ERSPs) to word presentation during sleep**.

Event-related changes in spectral power (ERSP) suggested that the ERP at 500 ms was accompanied by slow sleep spindles, while the ERP at 1000 ms was accompanied by fast spindles. Sleep spindles are NREM-sleep specific oscillatory events at frequencies between 9 and 16 Hz and with durations of 0.5–3 s (De Gennaro and Ferrara, 2003; Lüthi, 2013). They are separated into slow spindles (<12 Hz) that occur preferably during down-states and fast spindles (>12 Hz) that occur more frequently during up-states of both naturally occurring (Mölle et al., 2011, 2002) and entrained slow-oscillations (Ngo et al., 2013a,b). We found a significant word-induced power increase in the slow-spindle frequency band (see SS+ in Figure 2B) that co-occurred with the negative response at 500 ms, followed by a power increase (FS+) in fast spindle frequencies within the time window of the ERP at 1000 ms response. The FS+ was followed by a decrease (FS−) in fast spindle activity. These power changes in the spindle frequency range were significant at *p* < 0.01 (see Table 2 for descriptive statistics and Figure 4 for ERSP plots of all recorded electrodes). Because slow-oscillations are known to drive fast spindles to up-states and slow spindles to down-states (Mölle et al., 2011, 2002), the co-occurrence of the ERP at 500 ms with slow- and the ERP at 1000 ms with fast-spindle activity further indicates that the reported ERPs reflect slow-oscillations.

**Table 2:** **Descriptive statistics for the event-related changes in spectral power that were evoked by words presented during sleep**.

**^#^Response**	**Duration (ms)**		**Frequency (Hz)**		^*^p-Value	**Locus of maximal deviation from baseline**			
	**Start**	**End**	**Min**.	**Max**.		**Electrode**	**Time**	**Frequency**	***t*-Value**
SS+	340	928	4	11	0.001	Fz	562	5.5	10.5187
FS+	832	1722	8.5	21.5	0.002	F3	1294	15.5	7.1544
FS−	1706	2470	12	21.5	0.005	P4	1930	17.5	−5.5267

* *p-values are based on non-parametric statistics as suggested by Maris and Oostenveld (2007)*.

# *Responses: increases (+) and decreases (−) in slow- and fast-spindle (SS and FS respectively) frequencies*.

**Figure 5:**
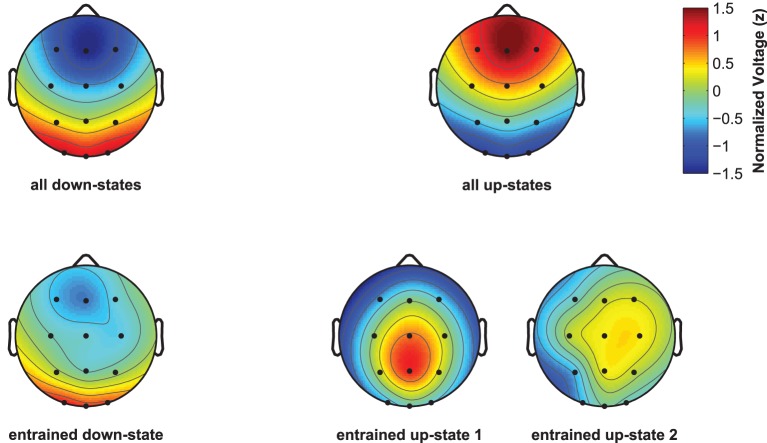
**Topographic plots for naturally occurring and entrained down- and up-states**. Topographies were normalized within each participant and were then averaged across participants. Plots display the topography at the peak of the ERP for all discretely identified down- and up-states (**top line**, left and right), and of the ERPs reflecting the entrained down-state and entrained up-states 1 and 2 (**bottom line**, left and right plots).

Although spontaneously occurring up-states showed the typical fronto-central maximum at electrode Fz, entrained up-states peaked at centro-parietal sites (see Figure 5). This was suggested by the analyses of normalized topographies of the peaks of all averaged spontaneous up-states and the peaks of entrained up-states 1 and 2. Repeated measures ANOVAs with the within-subject factors Topography (the 12 electrodes) and Event (spontaneous vs. entrained) revealed that the topographic differences were significant. This was suggested by the significant Topography X Event interactions if up-state 1 [*F*_(11, 165)_ = 24.20, *p* < 0.01, η^2^_*p*_ = 0.62] and up-state 2 [*F*_(11, 165)_ = 12.27, *p* < 0.01, η^2^_*p*_ = 0.45] were contrasted with spontaneously occurring up-states. Spontaneous and entrained down-states showed visually similar topographies with maximal negativity over fronto-central sites. However, the significant interaction term in the ANOVA still indicated that the topographies differed between spontaneous and entrained down-states [*F*_(11, 165)_ = 5.00, *p* < 0.01, η^2^_*p*_ = 0.25] because the difference between frontal and posterior negativity was less pronounced in the entrained compared to spontaneous down-states.

The analysis of ERSPs did not reveal a general increase in high-frequency activity that would be observed if sleep-played words had reduced sleep depth. Thus, word presentation did not awake our participants. To underscore this conclusion, we contrasted the mean spectral power following word-onset with the mean power of the pre-stimulus time window for the theta (4–8 Hz), alpha (4–12 Hz), sigma (11–16 Hz), beta (12–24 Hz), and gamma (>24–50 Hz) frequency band. Mean spectral activity was not significantly altered by word presentation in any of these bands [all *t*_(15)_ < 1.8, all uncorrected *p* > 0.09, all *r* < 0.42].

In sum, we have reasons to believe that word presentations during sleep did not wake our participants but entrained their slow oscillations and modulated their sleep spindle activity.

### Implicit retrieval of sleep-played words following waking

**Figure 6:**
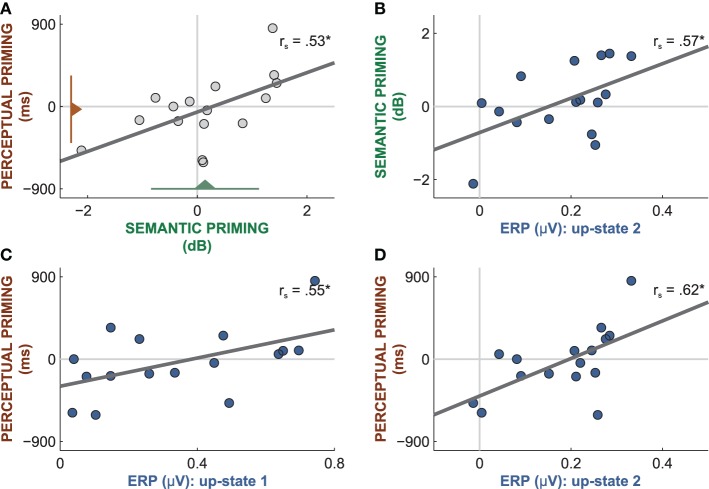
**Correlations between word-related brain responses and the amount of priming. (A)** Although the amount of priming was at chance level in both tests (triangles with lines reflect mean and standard deviation), perceptual and semantic priming were correlated. **(B)** The mean amplitude of the word-evoked up-state 1 predicted the amount of semantic priming. The mean amplitude of the word-evoked up-state 1 **(C)** and 2 **(D)** predicted the amount of perceptual priming. *r_s_*: Spearman correlations; ^*^p < 0.05.

The performance on both priming tests was at chance level for participants as a group (Figure 6A). Performance in the semantic priming test was expressed as the difference in identification volumes for new words [mean ± s.e.m. = 54.07 ± 0.58 dB(A)] minus synonyms to sleep-played words [53.89 ± 0.57 dB(A)]. This difference score did not significantly diverge from zero [*t*_(15)_ = 0.58, *p* = 0.57, *r* = 0.15]. The perceptual priming scores contained the difference in response latencies between incorrect word choices (2720 ± 232 ms) minus correct word choices (2750 ± 229 ms). Again, these scores did not significantly diverge from zero [*t*_(15)_ = −0.33, *p* = 0.75, *r* = −0.09]. Hence, participants showed no implicit memory for sleep-played words, when tested at the group-level.

However, performance on the two priming tests was linearly correlated between participants (*r*_s_ = 0.53, *p* = 0.04, Figure 6A), which hints at perceptual and semantic priming effects in some but not all participants. Because different subsets of sleep-played words were used in the two tests, the significant correlation is not a product of carry-over effects from the first to the second priming test. This significant correlation suggests that the priming scores reflect true interindividual cognitive differences rather than mere noise. These cognitive differences must mirror different degrees of word priming because success on both tests cannot be mediated by any other psychological or stimulus-inherent factor. Hence, those participants who yielded high priming scores on both tests must have been able to encode the percept and the meaning of sleep-played words. Moreover, the magnitude of word-entrained up-states predicted the amount of priming on both tests. Participants with larger mean amplitudes in up-state 1 and 2 performed better on both priming tests. The amplitude of up-state 2 correlated positively with perceptual priming (Figure 6D; *r*_s_ = 0.62, *p* = 0.01) and with semantic priming (Figure 6B; *r*_s_ = 0.57, *p* = 0.02). The amplitude of up-state 1 correlated positively with perceptual priming (Figure 6C; *r*_s_ = 0.55, *p* = 0.03), but not semantic priming (*r*_s_ = 0.03, *p* = 0.83). These findings suggest that up-states contributed to word encoding. It appears that those participants who consistently increased their neocortical excitability to a large extent in response to words were capable of forming enduring neural representations of words that facilitated priming following sleep. Note that the magnitude of the entrained down-state was not related to performance in either test (all |*r_s_*| < 0.36, all *p* > 0.17).

Which factors account for successful word encoding during sleep? We looked at various variables underlying sleep quality but none was predictive of the amount of priming in either test (all |*r_s_*| < 0.39, all *p* > 0.13). These variables were time spent in NREM sleep, number of words presented during sleep, average sleep depth during word presentation, the number of stops during word presentation due to arousals, mean amplitude of all detected up-states, and the average volume at which participants identified words in the semantic test. However, we found that the mean individual volume at which words were played during sleep was correlated with the amplitude of the entrained down-state (*r_s_* = −0.53, *p* = 0.04) and by trend with the amplitude of the first (*r_s_* = 0.46, *p* = 0.07) but not the second (*r_s_* = 0.23, *p* = 0.38) entrained up-state. This suggests that the entrainment of slow-oscillations was influenced by stimulus volume. However, the mean presentation volume was not correlated with semantic or perceptual priming (all |*r_s_*| < 0.22, all *p* > 0.40). Thus, presentation volume did not directly contribute to word encoding and to subsequent priming.

Words were not processed consciously during sleep. Participants asserted that they had not heard any words while asleep. Furthermore, choice accuracy reflecting conscious recognition in the perceptual forced-choice test was at the guessing level of 50% [mean ± s.e.m. = 49.03 ± 2.21%, *t*_(15)_ = −0.44, *p* = 0.68, *r* = 0.11]. Furthermore, choice accuracy was uncorrelated with up-state amplitude (all |*r_s_*| < 0.30, all *p* > 0.26), semantic priming (*r_s_* = −0.45, *p* = 0.08), and perceptual priming (*r_s_* = −0.06, *p* = 0.82). This is evidence that sleep-played words left no consciously accessible memory traces following waking.

## Discussion

To investigate whether humans can encode familiar everyday words during sleep, we played single words while participants were in NREM sleep during an afternoon nap. Words were presented rhythmically every 4 s which entrained sleep-specific slow-oscillations with up-states at around 1000 and 2400 ms following word onset. Following the nap, we administered a perceptual and a semantic priming test to tap participants' implicit memory for sleep-played words. Participants as a group performed at chance level in both priming tests. Nevertheless, some participants in the group must have encoded words during sleep as suggested by correlation analyses. Priming scores of both tests were positively correlated and were predicted by the magnitude of word-entrained up-states. Hence, participants with stronger entrainment of up-states—as reflected in larger word-related up-state amplitudes—showed stronger priming on both tests. These correlations indicate that some participants have encoded sleep-played words in a way that allowed them to reactivate the words' percepts and meanings following waking. The findings further suggest that up-states might have contributed to sleep-encoding. We conclude that the encoding of everyday words is feasible during sleep for some but not all individuals and benefits from up-states of slow-oscillations.

Sleep-played words were processed unconsciously and did not induce wakefulness as indicated by polysomnographic data recorded during sleep. Moreover, participants were unable to recognize sleep-played words following waking, which suggests that their sleep was not interrupted. EEG data of the polysomnogram suggested that word presentation entrained specific features of NREM sleep, namely slow-oscillations and spindles. The presence of slow-oscillations indicates that participants remained asleep and thus unconscious while words were being played for encoding. Unconsciousness of encoding was fostered by the low volume level used for word presentation. In addition, sleep-played words were partially masked by the constantly playing background noise. Following the nap, none of the participants reported having noticed words during sleep. Furthermore, conscious recognition of sleep-played words was at chance level and was neither correlated with the performance on the implicit memory measures nor with the size of the entrained up-states. Our behavioral data thus confirm the polysomnographic data suggesting complete unconsciousness of word encoding.

Polysomnographic data suggest that rhythmic presentation of words during sleep entrained a sequence of two slow-oscillations with up-states occurring at around 1000 and 2400 ms following word onset. The time delay of about 1.4 s between up-states indicates that entrained slow-oscillations appeared at a rate of 0.7 Hz. This rate reflects the characteristic frequency (0.7–0.8 Hz) of spontaneous slow-oscillations in humans (Achermann and Borbély, 1997). Previous studies reported entrainment of slow-oscillations at very similar rates (Ngo et al., 2013a,b). In these studies, noise sounds of 50 ms duration were played at rates of 0.8 or 0.93 Hz (one sound every 1.25 or 1.075 s), which entrained slow-oscillations at just these frequencies. Although we used two-syllabic nouns of varying duration and played them at a rate of 0.25 Hz (1 word every 4 s), entrained slow-oscillations still appeared at their characteristic rate of 0.7 Hz. This suggests that entrainment of slow-oscillations is possible using meaningful sounds and does not depend on the exact duration of sounds or on a presentation rate that lies within the slow-oscillations frequency range. We assume that entrainment is successful as long as sounds are presented at regular intervals that leave sufficient time for one or several full slow-oscillations to unfold between two consecutive stimuli. Random intervals (Ngo et al., 2013a) and intervals shorter than one slow-oscillation cycle (Ngo et al., 2013b) were indeed shown to interfere with slow-oscillation activity.

Auditory stimuli frequently evoke K-complexes in the EEG of NREM sleep but we think that word-evoked brain responses were not affected by these electroencephalographic events. K-complexes are marked by a strong hyperpolarization followed by a depolarization and are very similar to slow-oscillations regarding morphology and generating mechanism (Amzica and Steriade, 2002; Colrain, 2005; Cash et al., 2009). However, K-complexes are singular events that rarely occur in groups. Because slow-oscillations occur in groups of 2–3 cycles (Mölle et al., 2011), the long-lasting oscillatory activity (up-state 2) observed here is more likely to reflect slow-oscillations than K-complexes. Furthermore, evoked K-complexes are rare if stimuli are presented at high rates (once every 5 s), especially during deep NREM sleep (Bastien and Campbell, 1994). Slow-oscillations on the other hand are specific to deep sleep and are entrained by rhythmic and frequent stimuli (Ngo et al., 2013a,b). Because we presented words at high rates and targeted presentation to deep NREM sleep, we are confident that the reported brain responses were mainly driven by slow-oscillation activity. However, even if K-complexes contributed to evoked responses, the similarity between K-complexes and slow-oscillations suggests that the hyperpolarized phase of K-complexes could foster plastic synaptic changes in similar ways as up-states of slow-oscillations.

Word-entrained up-states seemed to have different neural generators than spontaneously occurring up-states. This was suggested by the finding that entrained up-states reached maximum around centro-parietal sites, whereas spontaneous up-states showed the typical topography with a maximum over fronto-central sites. Previous studies reported that sound-entrained and spontaneous up-states produce very similar topographies and might thus be generated by similar neuronal mechanisms (Ngo et al., 2013b). However, these studies used non-verbal sounds to entrain slow-oscillations. It is possible that the altered neural generators of word-entrained up-states reflect word-specific processing. But this interpretation remains speculative because our design entailed no control condition such as the play of non-sense words. Hence, a comparison between meaningful words and non-sense words cannot be computed to demonstrate a topographical difference due to semantic word processing.

Mean performance was at chance level in the two priming tests that probed implicit memory for sleep-played words. This makes believe that participants did not encode sleep-played words and showed no priming effects following sleep. However, performance on both priming tests was positively correlated with participants exhibiting higher semantic priming scores also exhibiting higher perceptual priming scores. Moreover, performance on both priming tests was correlated with word-evoked up-state amplitudes. The significant correlations between these three variables cannot be products of noise or chance. The relation between these three variables must have a biological origin. We assume that participants had encoded the sleep-played words to varying degrees with some participants showing positive priming and others negative priming. It should be noted that some participants displayed negative priming scores on both tests. Negative priming scores reflect impaired processing of the percepts and meanings of sleep-played words following waking. It is thus possible that successful encoding of words during sleep induced consistent positive priming in some and negative priming in other participants leading to zero net priming at the group level. This phenomenon is known from experiments on subliminal priming. Snodgrass and Shevrin (2006) found that a main effect of priming was absent because some participants showed positive priming, while others displayed negative priming due to differences in stimulus preferences and processing strategies. We assume that positive and negative priming are two natural consequences of encoding during sleep. According to Newman and Norman (2010), the direction of priming depends on the strength of activation of prime representations. The authors argued that strong activation of stimulus representations improves these representations and leads to positive priming, whereas weak activation impairs representations and yields negative priming. Their assumption is supported by the finding that stimuli which are masked or ignored and are thus only weakly activated induce negative priming (Ortells and Tudela, 1996; Noguera et al., 2007; Newman and Norman, 2010; Bermeitinger et al., 2012). Negative priming in our study was most pronounced in participants with minimal entrainment of up-states. We speculate that NREM sleep and especially down-states (Schabus et al., 2012) prevented strong activation of words' representations. Thus, the state of sleep might actually have impaired or weakened neuronal representations of percepts and concepts that were triggered by words—unless words were followed by up-states. We assume that the neuronal excitability and plasticity provided by up-states allowed a stronger activation of words during sleep, leading to a strengthening of words' representations. Because most participants displayed entrainment to some degree, the excitability provided by entrained up-states counteracted the weakening of word representations. In participants who consistently produced precisely timed large amplitude up-states upon word-onset, word activation was sufficient to even induce a strengthening of representations, leading to positive priming.

Performance on the priming tests administered after sleep was predicted by the magnitude of word-entrained up-states. Hence, up-states of slow-oscillations might have contributed to word encoding and to the strengthening of words' representations during sleep. Entrained up-states are not a specific indicator of word processing. In fact, up-states appear naturally, in the absence of any external triggers, and can be entrained by various non-verbal stimuli, such as pure tones (Ngo et al., 2013a,b), transcranial direct current stimulation (Marshall et al., 2006), and transcranial magnetic stimulation (Massimini et al., 2007). However, up-states reflect a brain-state that could serve as a window of opportunity for unconscious encoding. So far, slow-oscillations and up-states have only been associated with the consolidation of memories that were encoded before and not during sleep (Marshall et al., 2004, 2006; Ngo et al., 2013b). The sleeping brain repeatedly reactivates and replays the neuronal activity that represents memories learned before going to sleep (Skaggs and McNaughton, 1996; Ji and Wilson, 2007). This replay is thought to strengthen memory traces (Rudoy et al., 2009; Van Dongen et al., 2012) by inducing synaptic changes within the networks that represent the memory traces. Importantly, memory replay occurs predominantly during up-states of slow-oscillations (Ji and Wilson, 2007) because synaptic plasticity is increased during up-states. The plasticity provided by up-states should also contribute to the acquisition of new information during sleep. The coupling of words with up-states in the EEG suggests that the neuronal networks supporting word encoding were in a state of plasticity that allowed for long-term synaptic modifications. These synaptic changes may outlast the state of sleep to facilitate the perceptual and semantic processing of the same words or semantically related words following sleep. Evidence for this is provided by the finding of a larger entrainment of up-states in those participants, who yielded strong positive priming. It appears that participants who consistently produced large-amplitude up-states at 1000 and 2400 ms after word onset exhibited a facilitated perceptual and semantic processing of sleep-played words or their synonyms following sleep.

The excitability and plasticity provided by up-states could in principle assist both, the initial processing and the ensuing consolidation of sleep-played words. Our study design did not allow capturing the early ERPs that represent the initial sensory and semantic analysis of sleep-played words. The sensory component was obscured by different word lengths. The semantic component could not be isolated because the appropriate control condition (e.g., presentation of non-sense words) was lacking. But drawing from previous studies of sleep-played words, we assume that the initial perceptual analysis occurred around 100 ms following word onset (Perrin et al., 1999; 2002) and the semantic analysis around 400 to 700 ms following word onset (Brualla et al., 1998; Perrin et al., 1999; Daltrozzo et al., 2012). Thus, encoding was terminated before the onset of the first entrained up-state at about 800 ms. This indicates that up-states contributed rather to the consolidation than the initial encoding of the words' sounds and meanings. Interestingly, entrained up-states that followed early after word presentation (up-state 1) were predictive of perceptual priming, whereas later up-states (up-state 2) predicted both perceptual and semantic priming. This indicates that early up-states assisted the consolidation of appearance characteristics of words such as the rhythm or sound of words, while later up-states assisted the strengthening of word meaning.

We are not the first to postulate that humans might encode words during sleep (e.g., Fox and Robbin, 1952; Elliott, 1968; Levy et al., 1972; Bierman and Winter, 1989). However, previous reports of sleep-encoding lack convincing proof that participants were sleeping continuously during verbal stimulation (for reviews see Simon and Emmons, 1955; Hoskovec, 1966; Aarons, 1976; Eich, 1990). It is possible that encoding was mediated by brief phases of wakefulness in these studies. Studies that properly monitored the absence of wakefulness did not find evidence of sleep-encoding. It is likely that evidence of encoding was absent in these studies because stimuli were presented during REM instead of NREM sleep (Tani and Yoshii, 1970; Wood et al., 1992) or because explicit instead of implicit retrieval tests were used to assess memory for sleep-played contents (Emmons and Simon, 1956; Simon and Emmons, 1956; Koukkou and Lehmann, 1968; Lehmann and Koukkou, 1974). The sleep stage, during which information is presented, might determine the success of sleep-encoding. Because only NREM but not REM sleep is thought to actively contribute to memory consolidation (Diekelmann and Born, 2010; Deliens et al., 2013), only NREM sleep might provide the necessary conditions for the long-term storage of new information. Indeed, Arzi et al. (2012) found that sleep-learned tone-odor associations are retained into wakefulness if they were acquired during NREM rather than REM sleep. Yet, not only the sleep stage of encoding but also the type of retrieval test decides about the success of sleep-encoding. Sleep-formed memories are acquired in a state of unconsciousness and might therefore not be remembered consciously in explicit tests after waking up (Wood et al., 1992; Arzi et al., 2012). Therefore, implicit tests need to be applied as they are tailored to tap unconscious expressions of memories.

Although the correlations between word-entrained up-states and perceptual and semantic priming suggest that up-states contributed to the encoding of sleep-played words and their meanings, we recognize that our findings are not necessarily specific to verbal information. Up-states are sleep-specific brain states of increased neuronal activity that might benefit various cognitive processes and that can be entrained by various non-verbal stimuli (Marshall et al., 2006; Massimini et al., 2007; Ngo et al., 2013a,b). Thus, entrained up-states might also contribute to the encoding of non-verbal stimuli such as environmental sounds or simple melodies and might facilitate subsequent processing of these stimuli due to priming.

Encoding during sleep is not a pervasive phenomenon as suggested by the fact that only part of our participants displayed positive perceptual and semantic word priming. Because sleep quality and other external factors did not correlate with the size of priming, we speculate that perceptual or psychological factors might explain the difference between individuals regarding sleep-encoding. We found that the volume of sleep-played words tended to correlate with the magnitude of the first entrained up-state. If entrained up-states reflect stimulus intensity, they might also mirror participants' auditory sensitivity during sleep. Participants who are more sensitive to auditory stimuli during sleep might have responded with stronger entrainment of up-states upon word presentation which improved their ability to encode and consolidate the sleep-played words. Whether psychological factors such as motivation to learn during sleep or openness to experience affected entrainment of up-states and word-encoding remains elusive as we did not measure any psychological characteristics. Many more studies are necessary to pin down the factors that modulate encoding during sleep.

### Conflict of interest statement

The authors declare that the research was conducted in the absence of any commercial or financial relationships that could be construed as a potential conflict of interest.

## References

[B1] Aarons L. (1976). Sleep-assisted instruction. Psychol. Bull. 83, 1–40. 10.1037/0033-2909.83.1.1 1019279

[B2] Achermann P. Borbély A. A. (1997). Low-frequency (<1 Hz) oscillations in the human sleep electroencephalogram. Neuroscience 81, 213–222. 10.1016/S0306-4522(97)00186-3 9300413

[B3] Amzica F. Steriade M. (2002). The functional significance of K-complexes. Sleep Med. Rev. 6, 139–149. 10.1053/smrv.2001.0181 12531149

[B4] Arzi A. Shedlesky L. Ben-Shaul M. Nasser K. Oksenberg A. Hairston I. S.. (2012). Humans can learn new information during sleep. Nat. Neurosci. 15, 1460–1465. 10.1038/nn.3193 22922782

[B5] Bastien C. Campbell K. (1994). Effects of rate of tone-pip stimulation on the evoked K-Complex. J. Sleep Res. 3, 65–72. 10.1111/j.1365-2869.1994.tb00109.x 10607110

[B6] Bergmann T. O. Mölle M. Schmidt M. A. Lindner C. Marshall L. Born J.. (2012). EEG-guided transcranial magnetic stimulation reveals rapid shifts in motor cortical excitability during the human sleep slow oscillation. J. Neurosci. 32, 243–253. 10.1523/JNEUROSCI.4792-11.2012 22219286 PMC6621327

[B7] Bermeitinger C. Wentura D. Koppermann C. Hauser M. Grass B. Frings C. (2012). The direction of masked auditory category priming correlates with participants' prime discrimination ability. Adv. Cogn. Psychol. Univ. Finance Manag. Wars. 8, 210–217. 10.2478/v10053-008-0116-6 22956986 PMC3434682

[B8] Bierman D. Winter O. (1989). Learning during sleep: an indirect test of the erasure-theory of dreaming. Percept. Mot. Skills 69, 139–144.

[B9] Bowers J. S. (2003). An abstractionist account of masked and long-term priming, in Masked Priming: The State of the Art, eds Kinoshita S. Lupker S. J. (New York, NY: Psychology Press), 39–55.

[B10] Brualla J. Romero M. F. Serrano M. Valdizán J. R. (1998). Auditory event-related potentials to semantic priming during sleep. Electroencephalogr. Clin. Neurophysiol. 108, 283–290. 10.1016/S0168-5597(97)00102-0 9607517

[B11] Carlesimo G. A. (1994). Perceptual and conceptual priming in amnesic and alcoholic patients. Neuropsychologia 32, 903–921. 10.1016/0028-3932(94)90042-6 7969866

[B12] Cash S. S. Halgren E. Dehghani N. Rossetti A. O. Thesen T. Wang C.. (2009). The human K-complex represents an isolated cortical down-state. Science 324, 1084–1087. 10.1126/science.1169626 19461004 PMC3715654

[B13] Colrain I. M. (2005). The K-complex: a 7-decade history. Sleep 28, 255–273. 16171251 10.1093/sleep/28.2.255

[B14] Daltrozzo J. Claude L. Tillmann B. Bastuji H. Perrin F. (2012). Working memory is partially preserved during sleep. PLoS ONE 7:e50997. 10.1371/journal.pone.0050997 23236418 PMC3517624

[B15] De Gennaro L. Ferrara M. (2003). Sleep spindles: an overview. Sleep Med. Rev. 7, 423–440. 10.1053/smrv.2002.0252 14573378

[B16] Deliens G. Leproult R. Neu D. Peigneux P. (2013). Rapid eye movement and non-rapid eye movement sleep contributions in memory consolidation and resistance to retroactive interference for verbal material. Sleep 36, 1875–1883. 10.5665/sleep.3220 24293762 PMC3825437

[B17] Delorme A. Makeig S. (2004). EEGLAB: an open source toolbox for analysis of single-trial EEG dynamics including independent component analysis. J. Neurosci. Methods 134, 9–21. 10.1016/j.jneumeth.2003.10.009 15102499

[B18] Diekelmann S. Born J. (2010). The memory function of sleep. Nat. Rev. Neurosci. 11, 114–126. 10.1038/nrn2762 20046194

[B19] Duss S. B. Oggier S. Reber T. P. Henke K. (2011). Formation of semantic associations between subliminally presented face-word pairs. Conscious. Cogn. 20, 928–935. 10.1016/j.concog.2011.03.018 21481607

[B20] Eich E. (1990). Learning during sleep, in Sleep and Cognition, eds Bootzin R. R. Kihlstrom J. F. Schacter D. L. (Washington, DC: American Psychological Association), 88–108.

[B21] Elliott C. R. (1968). Extracts from an experimental study of the retention of auditory material presented during sleep, in Current Research in Hypnopaedia - A Symposium of Selected Literature, ed Rubin F. (London: MacDonald), 6–27.

[B22] Emmons W. Simon C. (1956). The non-recall of material presented during sleep. Am. J. Psychol. 69, 76–81. 10.2307/1418117 13302501

[B23] Fox B. H. Robbin J. S. (1952). The retention of material presented during sleep. J. Exp. Psychol. 43, 75–79. 10.1037/h0057555 14907994

[B24] Hauner K. K. Howard J. D. Zelano C. Gottfried J. A. (2013). Stimulus-specific enhancement of fear extinction during slow-wave sleep. Nat. Neurosci. 16, 1553–1555. 10.1038/nn.3527 24056700 PMC3818116

[B25] Henke K. Mondadori C. R. A. Treyer V. Nitsch R. M. Buck A. Hock C. (2003). Nonconscious formation and reactivation of semantic associations by way of the medial temporal lobe. Neuropsychologia 41, 863–876. 10.1016/S0028-3932(03)00035-6 12667523

[B26] Hoskovec J. (1966). Hypnopedia in the Soviet Union: a critical review of recent major experiments. Int. J. Clin. Exp. Hypn. 14, 308–315. 10.1080/00207146608412973 5341687

[B27] Iber C. Ancoli-Israel S. Chesson A. Quan S. F. (2007). The AASM Manual for the Scoring of Sleep and Associated Events: Rules, Terminology and Technical Specifications. 1st Edn Westchester, IL: American Academy of Sleep Medicine.

[B28] Ikeda K. Morotomi T. (1996). Classical conditioning during human NREM sleep and response transfer to wakefulness. Sleep 19, 72–74. 8650467 10.1093/sleep/19.1.72

[B29] Ji D. Wilson M. A. (2007). Coordinated memory replay in the visual cortex and hippocampus during sleep. Nat. Neurosci. 10, 100–107. 10.1038/nn1825 17173043

[B30] Koenig T. Kottlow M. Stein M. Melie-García L. (2011). Ragu: a free tool for the analysis of EEG and MEG event-related scalp field data using global randomization statistics. Comput. Intell. Neurosci. 2011, 1–14. 10.1155/2011/938925 21403863 PMC3049349

[B31] Koenig T. Melie-García L. (2010). A method to determine the presence of averaged event-related fields using randomization tests. Brain Topogr. 23, 233–242. 10.1007/s10548-010-0142-1 20376546

[B32] Koukkou M. Lehmann D. (1968). EEG and memory storage in sleep experiments with humans. Electroencephalogr. Clin. Neurophysiol. 25, 455–462. 10.1016/0013-4694(68)90155-7 4182599

[B33] Lehmann D. Koukkou M. (1974). Computer analysis of EEG wakefulness-sleep patterns during learning of novel and familiar sentences. Electroencephalogr. Clin. Neurophysiol. 37, 73–84. 10.1016/0013-4694(74)90246-6 4135449

[B34] Levy C. M. Coolidge F. L. Staab L. C. (1972). Paired associate learning during EEG-defined sleep: a preliminary study. Aust. J. Psychol. 24, 219 10.1080/00049537208255807

[B35] Lüthi A. (2013). Sleep spindles: where they come from, what they do. Neurosci. Rev. J. Bringing Neurobiol. Neurol. Psychiatry 20, 243–256. 10.1177/1073858413500854 23981852

[B36] Maris E. Oostenveld R. (2007). Nonparametric statistical testing of EEG- and MEG-data. J. Neurosci. Methods 164, 177–190. 10.1016/j.jneumeth.2007.03.024 17517438

[B37] Marshall L. Helgadóttir H. Mölle M. Born J. (2006). Boosting slow oscillations during sleep potentiates memory. Nature 444, 610–613. 10.1038/nature05278 17086200

[B38] Marshall L. Mölle M. Hallschmid M. Born J. (2004). Transcranial direct current stimulation during sleep improves declarative memory. J. Neurosci. 24, 9985–9992. 10.1523/JNEUROSCI.2725-04.2004 15525784 PMC6730231

[B39] Marsolek C. J. (2003). What is priming and why?, in Rethinking Implicit Memory, eds Bowers J. S. Marsolek C. J. (Oxford: Oxford University Press), 41–64.

[B40] Massimini M. Ferrarelli F. Esser S. K. Riedner B. A. Huber R. Murphy M.. (2007). Triggering sleep slow waves by transcranial magnetic stimulation. Proc. Natl. Acad. Sci. U.S.A. 104, 8496–8501. 10.1073/pnas.0702495104 17483481 PMC1895978

[B41] Mölle M. Bergmann T. O. Marshall L. Born J. (2011). Fast and slow spindles during the sleep slow oscillation: disparate coalescence and engagement in memory processing. Sleep 34, 1411–1421. 10.5665/sleep.1290 21966073 PMC3174843

[B42] Mölle M. Eschenko O. Gais S. Sara S. J. Born J. (2009). The influence of learning on sleep slow oscillations and associated spindles and ripples in humans and rats. Eur. J. Neurosci. 29, 1071–1081. 10.1111/j.1460-9568.2009.06654.x 19245368

[B43] Mölle M. Marshall L. Gais S. Born J. (2002). Grouping of spindle activity during slow oscillations in human non-rapid eye movement sleep. J. Neurosci. 22, 10941–10947. 12486189 10.1523/JNEUROSCI.22-24-10941.2002 PMC6758415

[B44] Newman E. L. Norman K. A. (2010). Moderate excitation leads to weakening of perceptual representations. Cereb. Cortex 20, 2760–2770. 10.1093/cercor/bhq021 20181622 PMC2951848

[B45] Ngo H.-V. V. Claussen J. C. Born J. Mölle M. (2013a). Induction of slow oscillations by rhythmic acoustic stimulation. J. Sleep Res. 22, 22–31. 10.1111/j.1365-2869.2012.01039.x 22913273

[B46] Ngo H.-V. V. Martinetz T. Born J. Mölle M. (2013b). Auditory closed-loop stimulation of the sleep slow oscillation enhances memory. Neuron 78, 545–553. 10.1016/j.neuron.2013.03.006 23583623

[B47] Noguera C. Ortells J. J. Abad M. J. F. Carmona E. Daza M. T. (2007). Semantic priming effects from single words in a lexical decision task. Acta Psychol. (Amst.) 125, 175–202. 10.1016/j.actpsy.2006.07.007 16950164

[B48] Ortells J. J. Tudela P. (1996). Positive and negative semantic priming of attended and unattended parafoveal words in a lexical decision task. Acta Psychol. (Amst.) 94, 209–226. 10.1016/0001-6918(95)00045-311248942

[B49] Perrin F. Bastuji H. Garcia-Larrea L. (2002). Detection of verbal discordances during sleep. Neuroreport 13, 1345–1349. 10.1097/00001756-200207190-00026 12151800

[B50] Perrin F. Garcıìa-Larrea L. Mauguière F. Bastuji H. (1999). A differential brain response to the subject's own name persists during sleep. Clin. Neurophysiol. 110, 2153–2164. 10.1016/S1388-2457(99)00177-7 10616121

[B51] Rasch B. Born J. (2013). About sleep's role in memory. Physiol. Rev. 93, 681–766. 10.1152/physrev.00032.2012 23589831 PMC3768102

[B52] Rechtschaffen A. Kales A. (1968). A Manual of Standardized Terminology, Techniques and Scoring System for Sleep Stages of Human Subjects. Washington, DC: Public Health Service, US Government Printing Office.

[B53] Ruch S. Markes O. Duss S. B. Oppliger D. Reber T. P. Koenig T.. (2012). Sleep stage II contributes to the consolidation of declarative memories. Neuropsychologia 50, 2389–2396. 10.1016/j.neuropsychologia.2012.06.008 22750121

[B54] Rudoy J. D. Voss J. L. Westerberg C. E. Paller K. A. (2009). Strengthening individual memories by reactivating them during sleep. Science 326, 1079. 10.1126/science.1179013 19965421 PMC2990343

[B55] Schabus M. Dang-Vu T. T. Heib D. P. J. Boly M. Desseilles M. Vandewalle G.. (2012). The fate of incoming stimuli during NREM sleep is determined by spindles and the phase of the slow oscillation. Front. Sleep Chronobiol. 3:40. 10.3389/fneur.2012.00040 22493589 PMC3319907

[B56] Siclari F. LaRocque J. J. Postle B. R. Tononi G. (2013). Assessing sleep consciousness within subjects using a serial awakening paradigm. Conscious. Res. 4, 542. 10.3389/fpsyg.2013.00542 23970876 PMC3747360

[B57] Simon C. Emmons W. (1955). Learning during sleep? Psychol. Bull. 52, 328–342. 10.1037/h0043733 13245899

[B58] Simon C. Emmons W. (1956). Responses to material presented during various levels of sleep. J. Exp. Psychol. 51, 89–97. 10.1037/h0043637 13295494

[B59] Skaggs W. E. McNaughton B. L. (1996). Replay of reuronal firing sequences in rat hippocampus during sleep following spatial experience. Science 271, 1870–1873. 10.2307/2889381 8596957

[B60] Snodgrass M. Shevrin H. (2006). Unconscious inhibition and facilitation at the objective detection threshold: Replicable and qualitatively different unconscious perceptual effects. Cognition 101, 43–79. 10.1016/j.cognition.2005.06.006 16289068

[B61] Steriade M. Nunez A. Amzica F. (1993). A novel slow (< 1 Hz) oscillation of neocortical neurons *in vivo*: depolarizing and hyperpolarizing components. J. Neurosci. 13, 3252–3265. 8340806 10.1523/JNEUROSCI.13-08-03252.1993 PMC6576541

[B62] Stuart G. P. Jones D. M. (1995). Priming the identification of environmental sounds. Q. J. Exp. Psychol. A 48, 741–761. 10.1080/14640749508401413 7568996

[B63] Stuart G. P. Jones D. M. (1996). From auditory image to auditory percept: facilitation through common processes? Mem. Cogn. 24, 296–304. 10.3758/BF03213294 8718764

[B64] Tani K. Yoshii N. (1970). Efficiency of verbal learning during sleep as related to the EEG pattern. Brain Res. 17, 277–285. 10.1016/0006-8993(70)90082-X 4312859

[B65] Tulving E. Schacter D. Stark H. (1982). Priming effects in word-fragment completion are independent of recognition memory. J. Exp. Psychol. 8, 336–342 10.1037/0278-7393.8.4.336

[B66] Van Dongen E. V. Takashima A. Barth M. Zapp J. Schad L. R. Paller K. A.. (2012). Memory stabilization with targeted reactivation during human slow-wave sleep. Proc. Natl. Acad. Sci. U.S.A. 109, 10575–10580. 10.1073/pnas.1201072109 22691500 PMC3387124

[B67] Woltz D. J. Shute V. J. (1995). Time course of forgetting exhibited in repetition priming of semantic comparisons. Am. J. Psychol. 108, 499–525 10.2307/1423070

[B68] Wood J. M. Bootzin R. R. Kihlstrom J. F. Schacter D. L. (1992). Implicit and explicit memory for verbal information presented during sleep. Psychol. Sci. 3, 236–239 10.1111/j.1467-9280.1992.tb00035.x

