# Supplementary material for: Word encoding during sleep is suggested by correlations between word-evoked up-states and post-sleep semantic priming
Source: Front Psychol. 2014 Nov 14;5:1319. doi: 10.3389/fpsyg.2014.01319 (PMC4231834; doi:10.3389/fpsyg.2014.01319)
Supplement: Supplementary file 1 [file Table1.PDF]

Supplementary Table 1: list of all word triplets with corresponding word frequencies, concreteness ratings, durations and syllable counts of spoken words.

	Words			Word Frequency			Concreteness (dummy coded)		Duration of spoken word (s)			Num. Syllables
LIST	Prime	Synonym	Distractor	Prime	Syn.	Distr.	Prime	Distr.	Prime	Syn.	Distr.	Syn.
A	bote	brieftraeger	bussard	4.500	4.990	4.489	1	1	0.539	0.936	0.692	3
A	eifer	tatendrang	esel	6.230	5.268	5.900	0	1	0.665	0.926	0.652	3
A	eisen	stahl	echo	6.900	7.606	7.239	1	0	0.616	0.671	0.510	1
A	faden	garn	frachter	6.881	3.829	5.971	1	1	0.735	0.441	0.695	1
A	kahlkopf	glatze	klumpfuss	2.833	5.472	2.833	1	1	0.855	0.566	0.834	2
A	kerker	gefaengnis	karpfen	4.394	8.984	4.812	1	1	0.632	0.865	0.685	3
A	kopie	duplikat	kaese	6.908	3.045	6.889	1	1	0.618	1.043	0.537	3
A	lanze	speer	leinen	5.352	6.991	5.617	1	1	0.671	0.669	0.712	1
A	loeffel	kelle	lotto	5.823	4.369	6.813	1	0	0.632	0.598	0.542	2
A	mauer	wand	muehe	8.161	8.418	8.352	1	0	0.539	0.516	0.590	1
A	skepsis	zweifel	skandal	7.805	9.341	8.420	0	0	0.843	0.727	0.868	2
A	talent	begabung	taxi	7.932	6.040	7.172	0	1	0.725	0.793	0.532	3
A	tanne	eiche	tuempel	4.595	5.762	4.762	1	1	0.552	0.560	0.618	2
A	treue	loyalitaet	tafel	7.028	6.583	7.080	0	1	0.508	1.029	0.646	5
B	anzug	frack	anruf	7.412	5.198	7.427	1	0	0.737	0.557	0.923	1
B	applaus	beifall	atom	7.919	7.963	5.252	0	0	0.743	0.717	0.676	2
B	beichte	gestaendnis	braten	5.011	7.582	5.384	0	1	0.608	1.027	0.652	3
B	besitz	eigentum	beweis	8.257	7.192	8.428	0	0	0.796	0.898	0.781	3
B	huelse	schale	hobel	3.135	6.238	2.398	1	1	0.636	0.614	0.608	2
B	kanne	krug	kirsche	4.585	6.290	4.454	1	1	0.476	0.490	0.591	1
B	lampe	leuchte	lunge	6.439	4.419	6.555	1	1	0.650	0.611	0.502	2
B	muecke	moskito	maehne	5.587	2.485	5.100	1	1	0.526	0.964	0.679	3
B	nixe	meerjungfrau	nomen	3.714	4.595	3.664	1	0	0.553	1.167	0.688	3
B	pranke	pfote	purpur	3.401	4.127	3.367	1	1	0.582	0.674	0.654	2
B	quelle	ursprung	quote	7.858	6.845	7.956	1	0	0.545	0.743	0.650	2
B	ritze	spalt	ruecksitz	3.738	5.438	5.165	1	1	0.522	0.695	0.743	1
B	ruhe	stille	rahmen	9.188	7.480	9.682	0	1	0.605	0.697	0.662	2
B	schiene	gleis	sofa	6.948	6.358	7.008	1	1	0.645	0.585	0.692	1
C	aufprall	kollision	aufschub	6.720	6.673	6.370	0	0	0.750	0.879	0.906	4
C	buckel	ruecken	buerste	5.700	8.940	4.344	1	1	0.542	0.594	0.654	2
C	buechse	dose	bruehe	4.585	6.161	5.447	1	1	0.532	0.567	0.556	2
C	delikt	straftat	dogma	5.063	7.030	5.485	0	0	0.615	1.056	0.563	2
C	festung	burg	feder	6.404	7.226	6.576	1	1	0.820	0.460	0.606	1
C	gurgel	kehle	gloeckner	3.951	6.148	4.190	1	1	0.588	0.593	0.605	2
C	hoehle	grotte	herde	6.400	4.543	6.144	1	1	0.549	0.509	0.589	2
C	junge	bub	japan	10.035	5.892	9.529	1	0	0.507	0.508	0.752	1
C	klavier	piano	kanal	7.315	6.066	7.109	1	1	0.634	0.552	0.725	3
C	kredit	darlehen	kabel	7.955	7.822	7.754	0	1	0.629	0.768	0.586	3
C	pforte	tor	prosa	4.997	9.843	5.826	1	0	0.637	0.400	0.668	1
C	schleuder	katapult	semmel	2.944	3.135	3.689	1	1	0.661	0.870	0.653	3
C	taifun	tornado	tresen	6.554	5.938	6.485	1	1	0.837	0.696	0.725	3
C	vortrag	referat	vorgang	7.823	5.733	8.032	0	0	0.810	0.800	0.747	3

	Words			Word Frequency			Concreteness (dummy coded)		Duration of spoken word (s)			Num. Syllables
**LIST**	**Prime**	**Synonym**	**Distractor**	**Prime**	**Syn.**	**Distr.**	**Prime**	**Distr.**	**Prime**	**Syn.**	**Distr.**	**Syn.**
D	beutel	sack	banner	6.033	7.050	6.001	1	1	0.606	0.467	0.460	1
D	biene	hornisse	bluse	5.273	2.303	5.357	1	1	0.507	0.807	0.631	3
D	brause	dusche	blutwurst	4.663	6.639	3.912	1	1	0.613	0.502	0.898	2
D	einfall	idee	eignung	5.541	9.575	5.948	0	0	0.714	0.517	0.613	2
D	frisoer	coiffeur	fossil	4.875	2.773	5.063	1	1	0.685	0.604	0.739	3
D	humor	komik	hammer	7.829	6.035	7.386	0	1	0.638	0.609	0.532	2
D	kaefig	gehege	kuppel	6.337	6.153	6.096	1	1	0.655	0.647	0.537	3
D	laden	geschaeft	liter	8.617	9.776	9.169	1	0	0.680	0.765	0.565	2
D	lenkung	steuerung	leber	6.186	6.640	6.495	0	1	0.729	0.815	0.551	3
D	lumpen	lappen	luefter	4.710	5.613	4.615	1	1	0.763	0.614	0.672	2
D	menge	anzahl	morgen	9.312	8.244	9.678	0	0	0.515	0.794	0.753	2
D	paddel	ruder	plural	4.369	6.921	4.277	1	0	0.482	0.554	0.655	2
D	pastor	prediger	plastik	6.815	6.323	6.579	1	0	0.601	0.743	0.805	3
D	scharnier	gelenk	sirup	4.357	5.220	4.304	1	1	0.669	0.657	0.684	2
E	anstrich	bemalung	argwohn	5.908	4.025	5.829	0	0	0.864	0.835	0.864	3
E	beule	delle	binde	4.454	5.429	4.382	1	1	0.507	0.420	0.505	2
E	gefecht	schlacht	geschirr	6.755	7.140	5.940	0	1	0.715	0.811	0.559	1
E	geige	violine	gletscher	6.172	5.704	6.384	1	1	0.602	0.881	0.562	4
E	gigant	riese	gestruepp	4.990	6.370	5.193	1	1	0.663	0.607	0.733	2
E	hocker	sessel	hupe	6.133	6.666	4.554	1	1	0.503	0.708	0.611	2
E	komplott	verschwoerung	kamel	5.513	6.880	5.328	0	1	0.749	1.033	0.637	3
E	kueste	ufer	kino	8.659	7.839	8.549	1	1	0.582	0.558	0.523	2
E	mitte	zentrum	mutter	10.250	9.270	10.171	0	1	0.536	0.706	0.518	2
E	muenze	banknote	miene	6.148	3.638	6.766	1	1	0.610	0.878	0.662	3
E	ratte	maus	rampe	5.081	7.142	6.174	1	1	0.593	0.619	0.644	1
E	ruebe	karotte	regung	4.190	3.526	5.112	1	0	0.571	0.723	0.677	3
E	seide	samt	schachtel	5.583	8.168	5.642	1	1	0.700	0.677	0.706	1
E	vorhang	gardine	vorrat	6.920	3.401	6.230	1	0	0.764	0.718	0.815	3
F	abfall	kehricht	anbau	6.691	2.639	7.152	1	0	0.614	0.770	0.749	2
F	chaos	durcheinander	chronik	8.199	7.180	6.019	0	0	0.953	0.915	0.750	4
F	chauffeur	fahrer	choral	6.026	9.535	4.174	1	0	0.725	0.621	0.625	2
F	feuer	brand	finger	9.096	8.922	8.119	1	1	0.582	0.492	0.628	1
F	huegel	berg	hose	7.008	8.573	7.363	1	1	0.595	0.499	0.587	1
F	koenig	herrscher	koerper	8.832	6.783	9.100	1	0	0.668	0.557	0.572	2
F	koeter	hund	kocher	3.892	8.431	3.970	1	1	0.573	0.504	0.548	1
F	kordel	schnur	knorpel	4.263	5.366	4.174	1	1	0.676	0.643	0.731	1
F	kratzer	schramme	kleber	5.924	3.784	5.617	1	1	0.600	0.652	0.711	2
F	magie	hexerei	magnet	6.310	4.431	5.525	0	1	0.547	0.784	0.752	3
F	party	fest	pflege	8.271	10.396	7.739	0	0	0.570	0.613	0.741	1
F	pflaume	zwetschge	pony	4.804	1.386	5.328	1	1	0.647	0.636	0.445	2
F	profil	silhouette	pokal	7.947	5.756	7.490	0	1	0.669	0.766	0.661	4
F	torte	kuchen	taste	5.303	7.277	5.338	1	1	0.577	0.604	0.587	2
