# Supplementary material for: Word encoding during sleep is suggested by correlations between word-evoked up-states and post-sleep semantic priming
Source: Front Psychol. 2014 Nov 14;5:1319. doi: 10.3389/fpsyg.2014.01319 (PMC4231834; doi:10.3389/fpsyg.2014.01319)
Supplement: Supplementary file 2 [file Table2.PDF]

October, 2014

Supplementary Table 2: Descriptive statistics for word frequencies across all word lists and word types (number of words per list: 14).

	**Prime**		**Synonym**		**Distracter**	
**LIST**	***M***	***SD***	***M***	***SD***	***M***	***SD***
A	6.096	1.520	6.193	1.838	6.168	1.510
B	5.942	1.962	5.872	1.512	5.846	2.009
C	6.175	1.760	6.511	1.616	6.213	1.534
D	6.066	1.529	6.376	2.032	6.063	1.679
E	6.197	1.559	6.086	1.801	6.161	1.491
F	6.612	1.628	6.461	2.591	6.222	1.556

Note: mean and SD of the natural logarithms of the word frequencies drawn from the Leipzig Wortschatz Lexicon (http://corpora.informatik.uni-leipzig.de/) are reported.
