# Supplementary material for: Word encoding during sleep is suggested by correlations between word-evoked up-states and post-sleep semantic priming
Source: Front Psychol. 2014 Nov 14;5:1319. doi: 10.3389/fpsyg.2014.01319 (PMC4231834; doi:10.3389/fpsyg.2014.01319)
Supplement: Supplementary file 3 [file Table3.PDF]

October, 2014

Supplementary Table 3: Descriptive statistics for the duration (s) of spoken words across all word lists and word types (number of words per list: 14).

	**Prime**		**Synonym**		**Distracter**	
**LIST**	***M***	***SD***	***M***	***SD***	***M***	***SD***
A	0.652	0.103	0.738	0.189	0.651	0.104
B	0.616	0.090	0.746	0.189	0.679	0.093
C	0.651	0.108	0.661	0.182	0.667	0.093
D	0.633	0.082	0.650	0.114	0.650	0.116
E	0.640	0.103	0.727	0.149	0.644	0.105
F	0.643	0.099	0.647	0.119	0.649	0.091
