# Supplementary material for: Word encoding during sleep is suggested by correlations between word-evoked up-states and post-sleep semantic priming
Source: Front Psychol. 2014 Nov 14;5:1319. doi: 10.3389/fpsyg.2014.01319 (PMC4231834; doi:10.3389/fpsyg.2014.01319)
Supplement: Supplementary file 4 [file Table4.PDF]

October, 2014

Supplementary Table 4: Number of concrete words for each word list and word type (total number of words per list: 14).

LIST	Prime & Synonym	Distracter
A	10	10
B	10	9
C	10	9
D	10	9
E	10	11
F	10	9
