# Supplementary material for: Word encoding during sleep is suggested by correlations between word-evoked up-states and post-sleep semantic priming
Source: Front Psychol. 2014 Nov 14;5:1319. doi: 10.3389/fpsyg.2014.01319 (PMC4231834; doi:10.3389/fpsyg.2014.01319)
Supplement: Supplementary file 5 [file Table5.PDF]

October, 2014

**Supplementary Table 5: Descriptive statistics for the number of syllables of synonyms (primes and distracters were two-syllabic).**

| | Synonym | |
|---|---|---|
| **LIST** | ***M*** | ***SD*** |
| A | 2.286 | 1.139 |
| B | 2.000 | 0.784 |
| C | 2.286 | 0.914 |
| D | 2.286 | 0.611 |
| E | 2.286 | 0.914 |
| F | 2.000 | 1.038 |
